# Tick-borne zoonoses and commonly used diagnostic methods in human and veterinary medicine

**DOI:** 10.1007/s00436-020-07033-3

**Published:** 2021-01-18

**Authors:** Andrea Springer, Antje Glass, Julia Probst, Christina Strube

**Affiliations:** Institute for Parasitology, Centre for Infection Medicine, University of Veterinary Medicine Hannover, Buenteweg 17, 30559 Hanover, Germany

**Keywords:** One Health, Zoonoses, Metazoonoses, Ticks, Tick-borne diseases, Lyme borreliosis, Diagnostics, Serology, PCR, ELISPOT

## Abstract

Around the world, human health and animal health are closely linked in terms of the One Health concept by ticks acting as vectors for zoonotic pathogens. Animals do not only maintain tick cycles but can either be clinically affected by the same tick-borne pathogens as humans and/or play a role as reservoirs or sentinel pathogen hosts. However, the relevance of different tick-borne diseases (TBDs) may vary in human vs. veterinary medicine, which is consequently reflected by the availability of human vs. veterinary diagnostic tests. Yet, as TBDs gain importance in both fields and rare zoonotic pathogens, such as *Babesia* spp., are increasingly identified as causes of human disease, a One Health approach regarding development of new diagnostic tools may lead to synergistic benefits. This review gives an overview on zoonotic protozoan, bacterial and viral tick-borne pathogens worldwide, discusses commonly used diagnostic techniques for TBDs, and compares commercial availability of diagnostic tests for humans vs. domestic animals, using Germany as an example, with the aim of highlighting existing gaps and opportunities for collaboration in a One Health framework.

## Tick-borne diseases in the One Health perspective

Ticks represent a major threat for human and animal health worldwide due to their vector function for a variety of zoonotic protozoan, bacterial and viral pathogens. These pathogens often circulate unnoticed in nature in enzootic tick-vertebrate cycles but may cause significant morbidity and mortality when spilling over to humans or domestic animals (Jahfari and Sprong 2016). For example, *Anaplasma phagocytophilum* mainly circulates between ticks and wildlife, but certain strains may cause granulocytic anaplasmosis in humans, dogs and horses as well as so-called tick-borne fever in domestic ruminants (Jaarsma et al. 2019). Similarly, small wild mammals constitute the main reservoir for tick-borne encephalitis virus (TBEV), which may cause neurologic disease in humans, as well as dogs and horses (Pfeffer and Dobler 2011). Additionally, domestic animals may represent an infection reservoir for tick-borne diseases (TBDs) in humans, such as cattle for *Babesia divergens* (Zintl et al. 2003) and dogs for *Ehrlichia canis* (Rar and Golovljova 2011).

Many tick species transmit zoonotic pathogens; however, some are exceptional due to their vector function for a number of different zoonotic pathogens. Thus, both the tick species infesting different hosts at the wildlife-domestic animal-human interface and the pathogens transmitted by them are of significant One Health importance. Among the particularly important tick vectors are *Ixodes ricinus*, *Ixodes persulcatus* and *Ixodes scapularis*, which belong to the so-called *Ixodes ricinus* complex, a group of 14 *Ixodes* species with almost worldwide distribution (Keirans et al. 1999; Xu et al. 2003). Ticks of the *I. ricinus* complex are confirmed vectors of zoonotic protozoa (*Babesia* spp.), a number of bacteria (e.g. *Borrelia* spp. and Rickettsiales) as well as three different flaviviruses (TBEV, Louping ill and Powassan virus). Furthermore, *Dermacentor andersoni*, *Dermacentor variabilis* and *Amblyomma americanum* are of particular One Health significance in North America (Sonenshine 2018) due to their vector function for a number of zoonotic bacterial (e.g. *Rickettsia* spp. and *Ehrlichia* spp.) and viral (e.g. Powassan and Heartland virus) pathogens.

While most zoonotic TBDs are transmitted by hard ticks, soft ticks may also play a role as vectors (Dantas-Torres et al. 2012). Several *Ornithodoros* spp. may transmit relapsing fever borreliae (Talagrand-Reboul et al. 2018), and this tick genus might be implicated in the transmission of *Coxiella burnetii* (Duron et al. 2015) and Alkhurma fever virus (Sawatsky et al. 2014).

### Tick-borne zoonotic protozoans

**Fig. 1:**
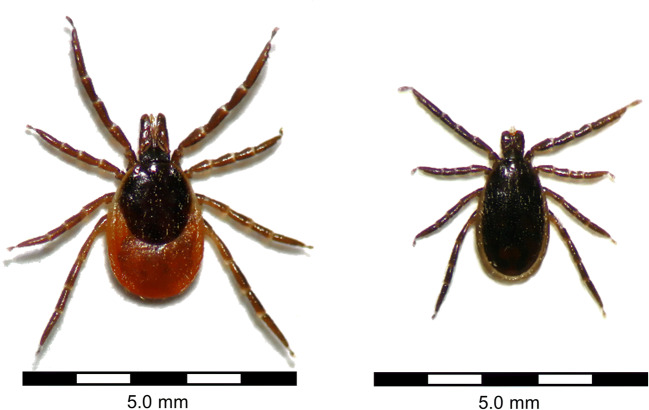
*Ixodes ricinus*, confirmed vector of *Babesia divergens*, *Babesia microti* and *Babesia venatorum*, among numerous other pathogens (left: female, right: male). Photographs were taken under an OPTIKA SLX-2 stereomicroscope (OPTIKA S.r.l., Ponteranica, Italy)

Among tick-borne pathogens, *Babesia* spp. constitute the only zoonotic protozoans (Table 1), which are transmitted to humans by *Ixodes ricinus* (Fig. 1) and *Ixodes scapularis* and are thus restricted to the range of these tick species in Eurasia, Northern Africa and North America. *Babesia* spp. are usually highly host-specific and the natural vertebrate hosts for *Babesia divergens*, *Babesia venatorum* and *Babesia microti* are cattle, wild ungulates and rodents, respectively, whereas humans are mainly affected if immunocompromised (Gray et al. 2010). Interestingly, although *Babesia microti* occurs in both Europe and North America, symptomatic human infections have so far only been acquired in North America (Azagi et al. 2020).

**Table 1:** Tick-borne protozoan pathogens, their vectors and reservoir hosts

Pathogen	Tick vector(s)^1^	Geographical distribution	Vertebrate reservoir(s)	Cell tropism in the vertebrate host	Comment(s)	References
*Babesia divergens*	*Ixodes ricinus*	Europe, North Africa, Russia	Cattle	Intracellular: erythrocytes		Reviewed by Zintl et al. (2003) and Gray et al. (2019b)
*Babesia microti*	*I. ricinus*, *Ixodes scapularis*	Eurasia, North America	Rodents	Intracellular: erythrocytes	So far, only North American strains involved in human cases	Reviewed by Gray et al. (2019b); Azagi et al. (2020)
*Babesia venatorum*	*I. ricinus*	Europe	Roe deer, possibly sheep	Intracellular: erythrocytes		Reviewed by Gray et al. (2019b); Gray et al. (2019a)

^1^Main tick vectors responsible for human infections; other tick vectors may be relevant in tick-reservoir cycles

### Tick-borne zoonotic bacteria

**Fig. 2:**
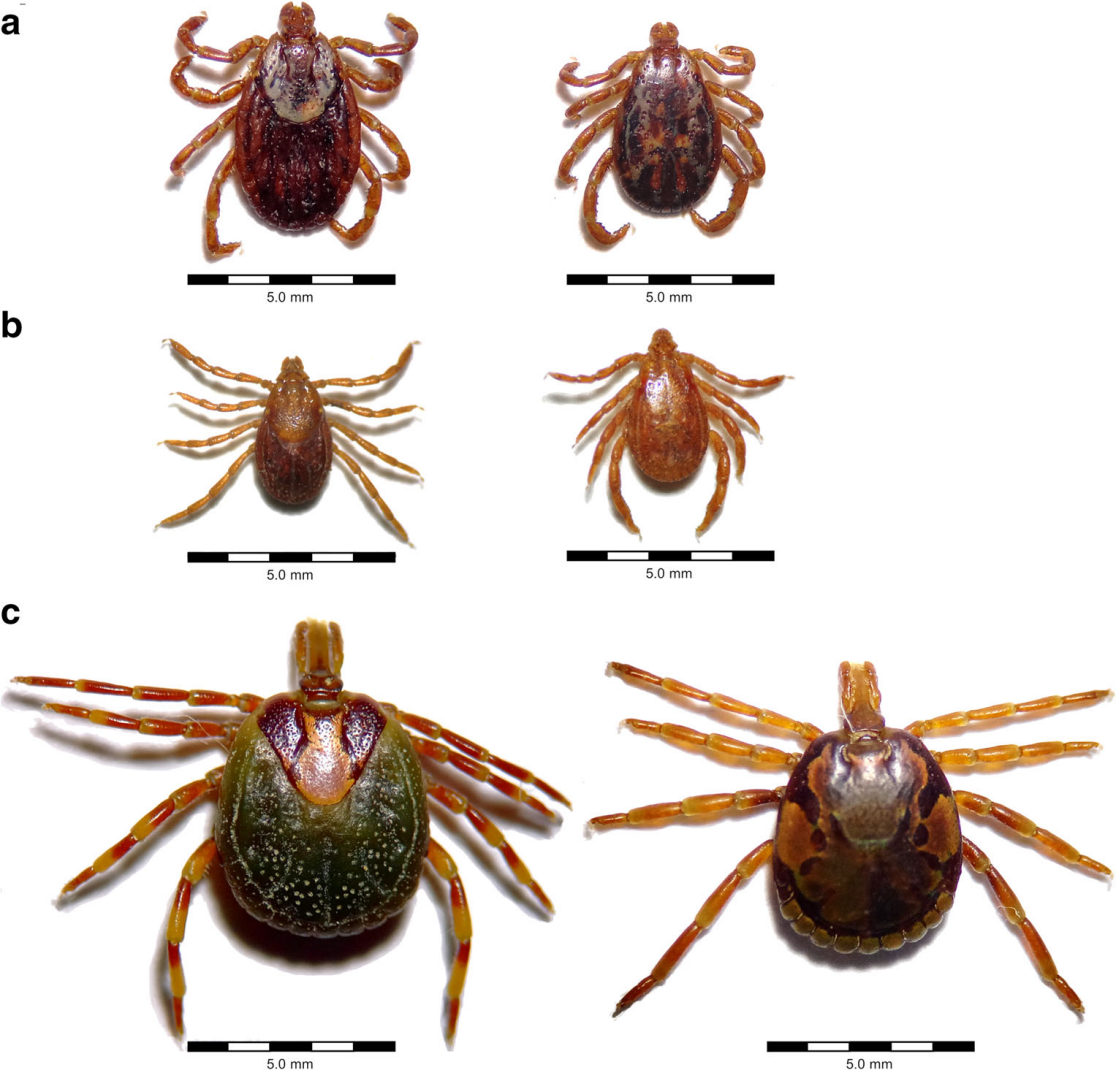
Important vectors of zoonotic tick-borne bacteria (left: females, right: males). **a** *Dermacentor variabilis*, confirmed vector of *Ehrlichia canis*, *Rickettsia rickettsii* and *F. tularensis*. **b** *Rhipicephalus sanguineus* s.l., confirmed vector of *E. canis*, *Rickettsia conorii* and *R. rickettsii*. **c** *Amblyomma hebraeum*, confirmed vector of *Ehrlichia ruminantium* and *Rickettsia africae*. Photographs were taken under an OPTIKA SLX-2 stereomicroscope (OPTIKA S.r.l., Ponteranica, Italy)

In contrast to protozoans, a wide variety of zoonotic bacterial pathogens are tick transmitted (Table 2). Some of these are of major importance due to their wide geographic distribution and/or the severity of the disease caused in humans and/or animals. For example, *Borrelia burgdorferi* sensu lato (s.l.), the causative agent of Lyme borreliosis, and *A. phagocytophilum* occur throughout the Northern Hemisphere as both are transmitted by ticks of the *Ixodes ricinus* complex. Furthermore, spotted fever group rickettsiae comprise a large group of species associated with zoonotic human disease or of unknown pathogenicity, which are transmitted by different species (Fig. 2) of several hard tick genera around the world (Parola et al. 2013). Some rickettsioses are associated with high case fatality rates in humans, especially Rocky Mountain spotted fever caused by *Rickettsia rickettsii* and transmitted mainly by *D. andersoni*, *D. variabilis* (Fig. 2a) and *Rhipicephalus sanguineus* s.l. (Fig. 2b), and Mediterranean spotted fever caused by *Rickettsia conorii* and transmitted mainly by *R. sanguineus* s.l. (Parola et al. 2013).

**Table 2:** Tick-borne bacterial pathogens, their vectors and reservoir hosts

Pathogen	Tick vector(s)^1^	Geographical distribution	Vertebrate reservoir(s)	Cell/tissue tropism in the vertebrate host	Comment(s)	References
Genus *Anaplasma*						
*Anaplasma phagocytophilum*	*Ixodes pacificus*, *Ixodes persulcatus*, *Ixodes ricinus*, *Ixodes scapularis*	Eurasia, North America	Zoonotic strains: red deer, possibly wild boar and hedgehogs	Intracellular: neutrophilic and eosinophilic granulocytes	Different strains with differing zoonotic potential	Jaarsma et al. (2019); Rar and Golovljova (2011)
*Anaplasma platys*	*Rhipicephalus sanguineus* s.s. (temperate lineage)	Worldwide	Dogs	Intracellular: thrombocytes	Rare human infections with unknown pathogenicity	Arraga-Alvarado et al. (2014); Snellgrove et al. (2020)
Genus *Bartonella*						
*Bartonella henselae* and other zoonotic *Bartonella* spp.	Probably^2^ *I. ricinus* and other ixodid ticks	Worldwide	Cats, rabbits, possibly dogs and rodents	Intracellular: erythrocytes	Predominantly other transmission routes^2^	Reviewed by Cheslock and Embers (2019)
Genus *Borrelia*						
Lyme borreliae: *Borrelia burgdorferi* sensu lato (s.l.) complex	*I. pacificus*, *I. persulcatus*, *I. ricinus*, *I. scapularis*	Eurasia, North America, South America	Small mammals, birds, lizards (depending on genospecies)	Extracellular: skin, joints, nervous system	Tissue tropism may differ between genospecies	Reviewed by Rudenko et al. (2011); Barbieri et al. (2013)
Relapsing fever borreliae: *Borrelia miyamotoi*	*I. ricinus*, *I. scapularis*, probably^3^ *I. pacificus*, *I. persulcatus*, *Ixodes ovatus*	Eurasia, North America	Small mammals	Extracellular: probably nervous system		Reviewed by Cutler et al. (2019)
Relapsing fever borreliae: *Borrelia duttonii*, *Borrelia hermsii*, *Borrelia turicatae* and others	*Ornithodoros* spp.	Asia, Africa, North America and South America	Small mammals	Extracellular: blood, different organs		Reviewed by Talagrand-Reboul et al. (2018)
Relapsing fever borreliae: *Borrelia lonestari*	*Amblyomma americanum*	North America	Deer	Extracellular: skin		Varela-Stokes (2007)
Genus *Coxiella*						
*Coxiella burnetii*	*Dermacentor andersoni*, *Dermacentor marginatus*, *Hyalomma asiaticum*, *Ixodes holocyclus*, *I. ricinus*, several *Ornithodoros* spp.	Worldwide	Ruminants	Intracellular: mononuclear phagocytes, pneumocytes, fibroblasts, endothelial cells	Transmission by inhalation of tick faeces more probable than by tick bite	Reviewed by Duron et al. (2015) and Voth and Heinzen (2007); Körner et al. (2020)
*Francisella tularensis*	*A. americanum*, *D. andersoni*, *D. marginatus*, *Dermacentor variabilis*, *I. ricinus*	Northern Hemisphere	Rodents and lagomorphs	Facultatively intracellular: macrophages, broad range of other cells	Multiple transmission routes, including bites of other arthropods	Reviewed by Telford III and Goethert (2020) and Ozanic et al. (2015); Výrosteková (1994)
Genus *Ehrlichia* and *Neoehrlichia*						
*Ehrlichia chaffeensis*	*A. americanum*, probably^3^ other tick species	North America, South America, Asia, Africa	Deer	Intracellular: monocytes/macrophages		Reviewed by Yabsley (2010) and Rar and Golovljova (2011)
*Ehrlichia canis*	*D. variabilis*, *Rhipicephalus sanguineus* tropical lineage	Worldwide	Canids	Intracellular: monocytes/macrophages	Rare human infections	Reviewed by Rar and Golovljova (2011); Moraes-Filho et al. 2015
*Ehrlichia ewingii*	*A. americanum*, probably^3^ other tick species	North America, South America, Africa	Deer	Intracellular: neutrophilic and eosinophilic granulocytes		Reviewed by Rar and Golovljova (2011)
*Ehrlichia muris euclairensis* (formerly *E. muris*–like)	*I. scapularis*	North America	Rodents	Intracellular: monocytes/macrophages	Rare human cases	Pritt et al. (2017); Karpathy et al. (2016)
*Ehrlichia ruminantium*	*Amblyomma hebraeum*, *Amblyomma variegatum* and other *Amblyomma* spp.	Africa	Domestic and wild ruminants	Intracellular: endothelial cells, neutrophilic granulocyte macrophages	Rare human cases	Reviewed by Rar and Golovljova (2011)
*Neoehrlichia mikurensis*	Probably^3^ *Ixodes ricinus* and other *Ixodes* spp.	Europe, Asia	Rodents	Intracellular: endothelial cells, neutrophilic granulocytes		Reviewed by Wennerås (2015); Wass et al. (2019)
Genus *Rickettsia*^4^						
*Rickettsia africae*	*A. hebraeum*, probably^3^ *A. variegatum* and other *Amblyomma* spp.	Africa, Caribbean	Unknown	Intracellular: endothelial cells, smooth muscle cells, monocytes/macrophages		Reviewed by Parola et al. (2013) and Sahni and Rydkina (2009); Kelly and Mason (1991)
*Rickettsia conorii*	*R. sanguineus* s.l., probably^3^ other species of the *Rhipicephalus sanguineus* group	Europe, Africa, Asia	Possibly dogs	Intracellular: endothelial cells, smooth muscle cells, monocytes/macrophages		Reviewed by Parola et al. (2013) and Sahni and Rydkina (2009)
*Rickettsia helvetica*	*I. ricinus*, *I. persulcatus*	Europe, North Africa, Asia	Small mammals	Intracellular: endothelial cells, smooth muscle cells, monocytes/macrophages		Reviewed by Parola et al. (2013) and Sahni and Rydkina (2009)
*Rickettsia rickettsii*	*A. americanum*, *Amblyomma aureolatum*, *Amblyomma cajennense*, *D. andersoni*, *D. variabilis*, *R. sanguineus* s.l.	North America, South America	Small mammals	Intracellular: endothelial cells, smooth muscle cells, monocytes/macrophages		Reviewed by Parola et al. (2013) and Sahni and Rydkina (2009)

^1^Main tick vectors responsible for human infections; other tick vectors may be relevant in tick-reservoir cycles

^2^Vector competence of ticks experimentally proven for *Bartonella birtlesii* only

^3^Vector competence not experimentally proven

^4^Only the most prevalent and/or pathogenic tick-borne *Rickettsia* spp. included; for an overview of other tick-borne *Rickettsia* spp. (see Parola et al. 2013)

**Fig. 3:**
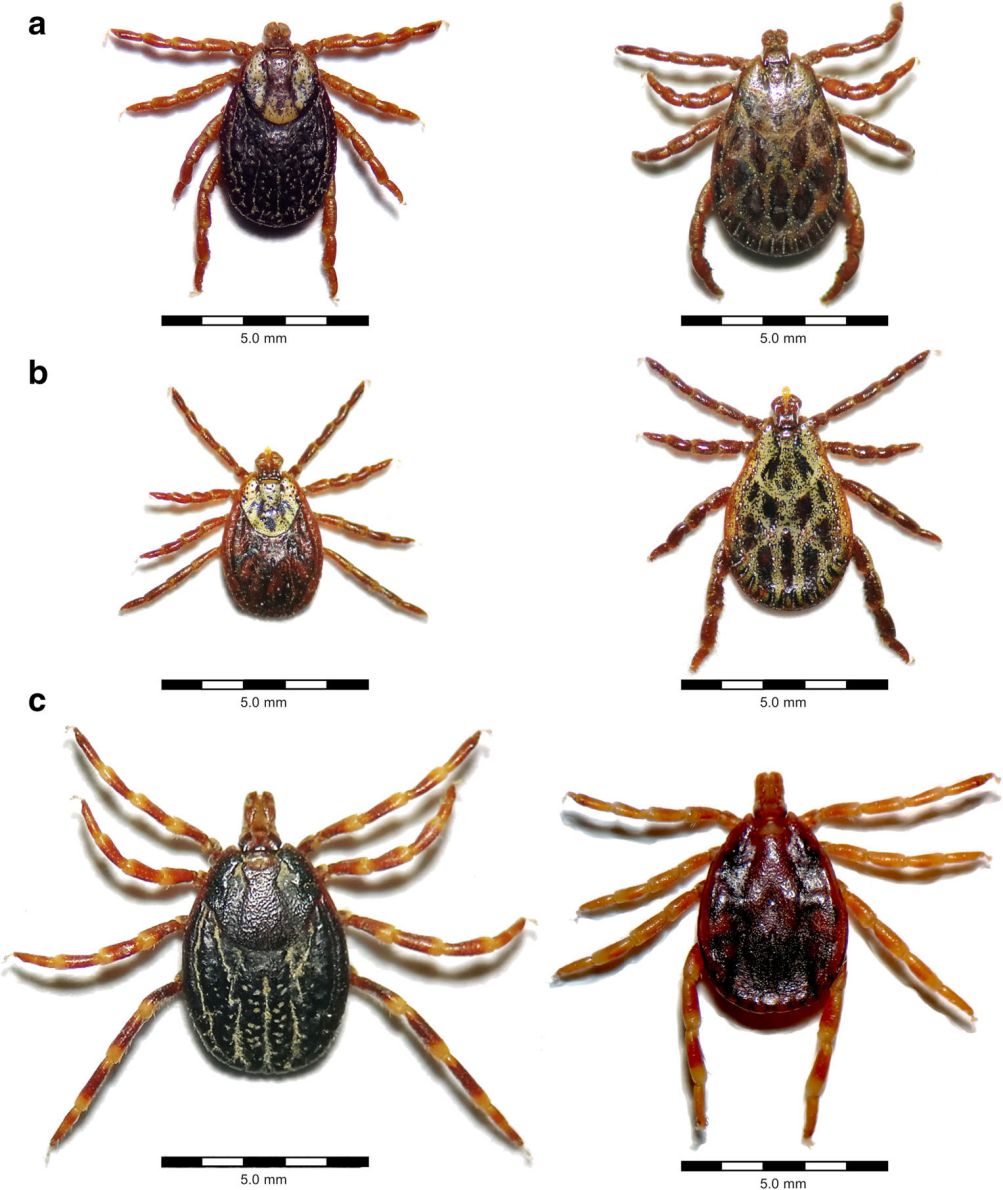
Important vectors of zoonotic tick-borne viruses (left: females, right: males). **a** *Dermacentor marginatus*, confirmed vector of Crimean-Congo haemorrhagic fever virus (CCHFV) and Omsk haemorrhagic fever virus (OHFV). **b** *Dermacentor reticulatus*, confirmed vector of OHFV and tick-borne encephalitis virus. **c** *Hyalomma rufipes*, confirmed vector of CCHFV. Photographs were taken under an OPTIKA SLX-2 stereomicroscope (OPTIKA S.r.l., Ponteranica, Italy)

In addition, ticks may play a role in the transmission of severe diseases such as tularemia, caused by *Francisella tularensis*, and so-called Q fever due to *C. burnetii* infection. Although other transmission routes are regarded as epidemiologically more important, several hard tick species, including *D. andersoni* in North America and *I. ricinus* (Fig. 1) as well as *Dermacentor marginatus* (Fig. 3a) in Eurasia, have been identified as competent vectors for both of these pathogens (Telford III and Goethert 2020; Duron et al. 2015).

### Tick-borne zoonotic viruses

Compared to bacteria, none of the tick-borne viruses are distributed worldwide (the same applies to protozoans, cf. Table 1), but rather often restricted to particular geographic regions (Table 3). However, many of them cause life-threatening disease in humans. Among tick-borne viruses, the highly pathogenic Crimean-Congo haemorrhagic fever virus (CCHFV), transmitted mainly by *Hyalomma* spp. (Fig. 3c), has the widest distribution as it occurs in Africa, throughout Asia and in Eastern Europe (IZS “G. Caporale” 2009). Likewise, TBEV has a rather wide distribution, with different subtypes circulating in *Ixodes* ticks in Europe, Siberia and far-eastern Asia (Dobler et al. 2012). Examples of highly pathogenic tick-borne viruses with a more restricted geographical distribution include Omsk haemorrhagic fever virus, transmitted by *D. marginatus* (Fig. 3a) and *D. reticulatus* (Fig. 3b) (Růžek et al. 2010) in Russia, and Kyasanur Forest virus, transmitted by *Haemaphysalis spinigera* (Shah et al. 2018) in India.

**Table 3:** Tick-borne viral pathogens, their vectors and reservoir hosts

Pathogen	Tick vector(s)^1^	Geographical distribution	Vertebrate reservoir (s)	Cell tropism in the vertebrate host	Comment(s)	References
Alkhurma virus	Unknown (possibly *Hyalomma* spp. or *Ornithodoros* spp.)	Saudi Arabia	Unknown	Unknown; probably mesangial cells, mononuclear phagocytes		Tambo and El-Dessouky (2018); Sawatsky et al. (2014)
Colorado tick fever virus	*Dermacentor andersoni*	North America	Small mammals	Haematopoietic cells		Reviewed by Yukl and Wong (2016)
Crimean-Congo haemorrhagic fever virus	*Dermacentor marginatus*, *Hyalomma impeltatum*, *Hyalomma marginatum*, *Hyalomma truncatum*, *Hyalomma rufipes*, *Rhipicephalus rossicus*	Southern Europe, Africa, Asia	Lagomorphs, large wild and domestic mammals	Mononuclear phagocytes, endothelial cells, hepatocytes		Reviewed by IZS “G. Caporale” (2009)
Heartland virus	*Amblyomma americanum*	North America	Unknown	Mononuclear phagocytes		Reviewed by Brault et al. (2018)
Kyasanur Forest disease virus	*Haemaphysalis spinigera*	India	Small mammals	Possibly monocytes/macrophages and dendritic cells		Reviewed by Shah et al. (2018)
Louping ill virus	*Ixodes ricinus*	British Isles, Norway, Spain	Sheep, lagomorphs, birds	Neurons, histiocytes	Rare human infections	Reviewed by Gilbert (2016); Sheahan et al. (2002)
Omsk haemorrhagic fever virus	*Dermacentor reticulatus*, *Dermacentor marginatus*	Russia	Small mammals	Haematopoietic and vascular tissues		Reviewed by Růžek et al. (2010)
Powassan virus/deer tick virus	*Dermacentor andersoni*, *Ixodes scapularis*, probably^2^ *Ixodes cookei*	North America, Russia	Small mammals	Neurons		Reviewed by Ebel (2010)
Severe fever with thrombocytopenia syndrome virus	*Haemaphysalis longicornis*	East Asia	Unknown, possibly domestic ruminants	Monocytes/macrophages, dendritic cells, B cells		Reviewed by Mansfield et al. (2017); Cheng et al. (2019); Suzuki et al. (2020)
Tick-borne encephalitis virus	*Ixodes persulcatus*, *I. ricinus*, *Ixodes ovatus*, *D. reticulatus*	Eurasia	Small mammals	Dendritic cells, neurons, glial cells		Reviewed by Dobler et al. (2012); Fares et al. (2020); Ličková et al. (2020)

^1^Main tick vectors responsible for human infections; other tick vectors may be relevant in tick-reservoir cycles

^2^Vector competence not experimentally proven

### Human and veterinary relevance of tick-borne zoonotic pathogens

The relevance of different tick-borne pathogens varies in the fields of human vs. veterinary medicine. For example, tick-borne encephalitis (TBE) cases occur mostly in humans and only rarely in domestic animals, which are mainly regarded as sentinels of virus occurrence (Imhoff et al. 2015). However, domestic ruminants are epidemiologically important as sources of alimentary human TBEV infections (Dobler et al. 2012) and dogs as well as horses may develop severe neurological signs when contracting TBE (Pfeffer and Dobler 2011; Waldvogel et al. 1981). Regarding the numerous tick-transmitted *Rickettsia* spp., which are relevant globally as agents of human disease (Parola et al. 2013), evidence of pathogenicity in domestic animals is limited to *Rickettsia conorii* and *Rickettsia rickettsii* in dogs (Keenan et al. 1977; Solano-Gallego et al. 2006).

In contrast, *B. divergens* is primarily a parasite of cattle, causing haemolytic anaemia with high case fatality rates in naïve cattle herds (Springer et al. 2020; Zintl et al. 2003), whereas human *B. divergens* cases mainly involve splenectomised or immunosuppressed patients (Azagi et al. 2020). Nevertheless, cases in immunocompetent persons have also recently been reported (Martinot et al. 2011). Similarly, *Ehrlichia canis* is of major veterinary relevance as the causative agent of canine monocytic ehrlichiosis, whereas human ehrlichiosis cases due to this pathogen are very rare (Rar and Golovljova 2011). Similarly, *A. phagocytophilum* is a frequent cause of disease in dogs, horses and ruminants in Europe (Silaghi et al. 2011; Kohn et al. 2008), whereas human cases are rarely reported on the continent (Azagi et al. 2020). In North America on the other hand, human granulocytic anaplasmosis cases are numerous but tick-borne fever in ruminants has never been confirmed (Dugat et al. 2015). These epidemiological differences are attributed to different circulating strains of *A. phagocytophilum* (Dugat et al. 2015).

Finally, Lyme borreliosis is sometimes (subjectively) regarded as equally important in both fields, especially by dog owners, although pathogenicity for dogs has only been proven for *B. burgdorferi* sensu stricto (s.s.) and remains questionable for other genospecies of the *B. burgdorferi* s.l. complex (Littman et al. 2018).

## Commonly used diagnostic methods for TBDs in human and veterinary medicine

The differences in clinical relevance of zoonotic TBDs are reflected by the availability of commercially manufactured human vs. veterinary diagnostic tests. However, as TBDs gain importance in both fields and rare zoonotic pathogens, such as *Babesia* spp., are increasingly identified as causes of human TBDs, a One Health approach in TBD diagnostics may lead to synergistic benefits. In the following, commonly used diagnostic techniques for TBDs in both fields and comparison of commercial availability of tests for humans vs. domestic animals are discussed, with the aim of highlighting gaps and opportunities for collaboration between medical and veterinary scientists.

### Direct detection methods

Traditionally, microscopy, culture of the pathogen or xenodiagnosis was widely used for direct detection of tick-borne pathogens in patient samples, but nowadays, nucleic acid–based methods are more commonly employed. Nevertheless, microscopic examination of stained blood smears is still the method of first choice for diagnosis of acute *Babesia* infections, in both human and veterinary medicine (Ord and Lobo 2015; Solano-Gallego et al. 2016). Furthermore, blood smear analysis is helpful to demonstrate intracellular morulae during anaplasmosis and ehrlichiosis (Schotthoefer et al. 2013). This method is relatively fast and low-cost; however, sensitivity depends on the level of parasitaemia and pathogen species differentiation is not always possible (Ord and Lobo 2015). Therefore, diagnosis should be corroborated by molecular techniques (Solano-Gallego et al. 2016).

Pathogen culture can be difficult and time consuming, may require special biosafety conditions and is therefore often performed by specialised laboratories only. Many tick-borne pathogens grow slowly and require special media or cell cultures. For example, the time to positive culture may span several weeks for *B. burgdorferi* s.l. (Eldin et al. 2019) and up to 30 days for *Rickettsia* spp. (Portillo et al. 2017). Challenges associated with culturing tick-borne pathogens are further illustrated by the example of *Neoehrlichia mikurensis*, which was only recently successfully cultured in human and tick cell lines, although the pathogen has been known since 2004 (Wass et al. 2019).

Nucleic acid amplification techniques are often more sensitive than the aforementioned methods and considerably faster than pathogen culture, improving diagnostic efficiency (Korber et al. 2017). In routine diagnostic settings, real-time quantitative PCR (qPCR) is often used due to increased sensitivity and speed as compared to conventional PCR. Additionally, real-time qPCR allows quantification by the gene copy numbers of the given pathogen or cycle threshold (Ct) values and can therefore also be useful for monitoring the course of infection (Che et al. 2019). However, it should be kept in mind that detection of DNA does not necessarily indicate that viable pathogens are present, and false-positive results may be obtained after successful treatment (Kuleš et al. 2017).

Adaptations of the real-time qPCR method include digital PCR (dPCR), which allows detection and quantification of rare target sequences by partitioning the sample into many parallel PCR reactions, thus improving test sensitivity. This technique has recently been successfully applied for *B. burgdorferi* s.l. identification in patient blood, which was previously hindered by extremely low numbers of circulating spirochaetes (Das et al. 2020).

Aside from singleplex PCRs, multiplex assays may be used as screening tests. For example, multiplex assays combining real-time qPCR detection of *A. phagocytophilum* with *Ehrlichia* spp. or *B. burgdorferi* s.l. are available (e.g. Courtney et al. 2004; Reller and Dumler 2018), while a broad-panel system for the simultaneous detection of nine tick-borne pathogens is currently available for research use only (Buchan et al. 2019). For patients suspected of sepsis, multiplex real-time qPCRs for simultaneous amplification of a wide range of pathogens have been developed (Guido et al. 2016); however, not all of them detect tick-borne pathogens. Recently, multiplex PCR followed by electrospray ionisation mass spectrometry (PCR/ESI-MS) has been used to diagnose early *B. burgdorferi* s.s. (Eshoo et al. 2012), *Ehrlichia* spp. and *R. rickettsii* (Eshoo et al. 2010) as well as *A. phagocytophilum* (Lagler et al. 2017) infections. This technique provides the advantage of identifying and genotyping pathogens in a short time, but it was only adopted by a few hospitals in Europe and was discontinued by the manufacturer in 2017, probably due to economic reasons (Özenci et al. 2017).

In general, PCR requires expensive equipment, which may be a problem in less-developed countries or in field settings. Loop-mediated isothermal amplification (LAMP) is a low-cost DNA amplification technique that works at a constant temperature and thus does not require a thermocycler (Becherer et al. 2020). LAMP assays to detect tick-borne pathogens have mainly been developed not only for veterinary applications (e.g. Faggion et al. 2013; Singh et al. 2019; Wang et al. 2017) but also for detection of TBEV (Hayasaka et al. 2013) and severe fever with thrombocytopenia syndrome virus (SFTSV) (Baek et al. 2018) in human patients in resource-limited settings.

Mass spectrometry–based approaches, e.g. matrix-assisted laser desorption ionisation time-of-flight (MALDI-TOF), are routinely used to identify cultured pathogens in microbiological laboratories, based on comparison of protein signatures to existing databases. Although not yet routinely used for diagnosis of TBDs, applicability for identification and typing of cultured *B. burgdorferi* s.l. has recently been demonstrated (Neumann-Cip et al. 2020). Mass spectrometry also offers new opportunities to identify biomarkers of specific diseases in patient samples, as shown, for example, for *Babesia microti* infections in an experimental hamster model (Magni et al. 2020). Similarly, MALDI-TOF analysis of canine serum samples may aid in the diagnosis of *Babesia canis* infections in dogs (Adaszek et al. 2014).

### Indirect detection methods: detection of humoral immune response

In some TBDs, direct pathogen detection is particularly difficult. For example, *B. burgdorferi* s.l. spirochaetes are only present at transient and low levels in patient blood (Schutzer et al. 2018). Similarly, direct detection of TBEV is only possible in the early, viraemic phase of the disease (Girl et al. 2020). Therefore, serological tests are commonly employed in TBD diagnosis. However, it has to be kept in mind that there is usually a time lag of several days to weeks between disease onset and development of antibody and, furthermore, that elevated antibody levels indicate pathogen exposure, but not necessarily current infection. Therefore, positive titres should always be interpreted in conjunction with the clinical presentation (Portillo et al. 2017; Sanchez et al. 2016). Acute infections may be detected by seroconversion or a rise in antibody titres. Therefore, testing of sequential samples taken several weeks apart is often recommended (e.g. Portillo et al. 2017; Solano-Gallego et al. 2016). IgM antibody titres are the first to rise and may therefore be targeted during early phases of the infection. However, IgM antibody tests are particularly prone to produce false-positive results and should thus be accompanied by other methods, e.g. direct pathogen detection or documentation of IgG seroconversion (Landry 2016; Seriburi et al. 2012). IgG avidity testing represents an additional approach to determine the stage of an infection, as IgG binding avidity increases as the infection progresses. For TBE, IgG avidity testing may be useful to rule out false-positive results due to cross-reactive IgM antibodies induced by other flaviviruses or in cases of atypical antibody responses, e.g. when IgM antibodies are persistently elevated past the acute phase of infection (Vilibic-Cavlek et al. 2016). For Lyme borreliosis, a recently developed IgG avidity Western blot has shown promising first results to identify disease stage (Mavin et al. 2018).

The most frequently used serologic methods include the enzyme-linked immunosorbent assay (ELISA), immunofluorescence antibody test (IFAT) and immunoblotting. ELISA tests can be performed with high sample throughput but may suffer from lower specificity as compared to other tests. Therefore, a two-tiered approach is often recommended, confirming positive or borderline ELISA tests with more specific techniques such as immunoblotting (e.g. in Lyme borreliosis, Sanchez et al. 2016) or seroneutralisation tests (e.g. in TBE, Reusken et al. 2019).

Modifications of the ELISA technique include magnetic bead–based multianalyte assays, which are characterised by high sensitivity even if antibody titres are low. Bead-based assays have been developed, for example, for the detection of anti-*B. burgdorferi* s.l. antibodies in humans (Gerritzen and Brandt 2012) as well as in horses and dogs (Wagner et al. 2011a; Wagner et al. 2011b).

For rickettsial diseases, the IFAT is considered the serological reference method (Portillo et al. 2017). IFATs are also commonly employed to detect and quantify anti-*Babesia* (Sanchez et al. 2016; Solano-Gallego et al. 2016) as well as anti-*Ehrlichia* antibodies (Dumler et al. 2007). However, the technique is relatively labour intensive as compared to ELISA and can be somewhat subjective as it involves microscopic evaluation of antigen-coated glass slides.

In addition, rapid immunochromatographic tests are commercially available for non-laboratory settings. These tests are easy to use; however, they offer only a positive/negative result, allowing no quantification of antibody titres. Furthermore, some commercially available rapid tests suffer from low sensitivity, as shown e.g. for Lyme borreliosis (Liu et al. 2018; Smit et al. 2015).

Sensitivity and specificity of serologic tests greatly depend on the antigen(s) used. Use of purified or recombinant antigens as well as synthetic peptides rather than whole-cell lysates may improve specificity. For example, ELISA tests based on a synthetic C6 peptide, a highly invariant region of the *B. burgdorferi* s.l. VlsE (variable major protein-like sequence, expressed) protein, have superior specificity as opposed to whole-cell antigen ELISAs (Waddell et al. 2016). However, cross-reactivity with sera from *Borrelia miyamotoi*–infected patients has recently been described (Molloy et al. 2017). In dogs, for which *B. burgdorferi* s.s. and s.l. (*Borrelia afzelii* and *Borrelia garinii*) vaccines are available, use of the C6 peptide in serological tests allows discrimination between vaccinated and infected animals (Pantchev et al. 2015). In human TBDs, discrimination between infection-induced and vaccination-induced antibodies is relevant for TBE. For this purpose, an ELISA based on the non-structural protein 1 (NS1) of TBEV has recently been developed, which is exclusively indicative of natural infection and also allows significant discrimination from other flavivirus infections (Girl et al. 2020).

Similar to direct tests, serological assays such as immunoblots and rapid immunochromatographic tests are also available in multiplex formats. For example, a rapid test frequently employed in veterinary medicine allows the simultaneous detection of canine antibodies against *B. burgdorferi* s.l., *Ehrlichia* spp. and *Anaplasma* spp., in addition to canine heartworm antigen (Chandrashekar et al. 2010).

### Indirect detection methods: detection of cellular immune response

Aside from antibody production, many tick-borne pathogens induce specific T cell responses. T cell–based assays might be helpful to bridge the gap between infection and onset of antibody production or might be employed as confirmatory tests to rule out false-positive serology results (Jin et al. 2013). The enzyme-linked immunospot assay (ELISPOT) is a sensitive method to measure the cytokine response of T cells upon antigen stimulation (Kalyuzhny 2005). ELISPOT assays have been developed for a variety of TBDs; however, their utility is controversially discussed, especially regarding Lyme borreliosis. ELISPOT assays developed for Lyme borreliosis, which exclusively measure interferon-γ release, show a wide range of sensitivity and specificity and poor reproducibility and are therefore currently not recommended for routine diagnostic use (Raffetin et al. 2020). Similarly, lymphocyte transformation tests (LTTs) assess the proliferative response of T cells upon stimulation with specific antigens. LTTs are offered by some laboratories for diagnosis of active Lyme borreliosis in humans; however, current guidelines do not recommend these tests due to low specificity (Dessau et al. 2014).

Cytokines and chemokines as evidence of a cellular immune response may also be measured directly in patient samples. For example, the chemokine CXCL13 in cerebrospinal fluid constitutes a sensitive and specific marker of acute Lyme neuroborreliosis in humans (Raffetin et al. 2020).

## Relevant zoonotic TBDs and commercial availability of diagnostic test kits by the example of Germany

In Germany, as in other central European countries, *I. ricinus* is the most relevant vector of zoonotic tick-borne pathogens, including *B. burgdorferi* s.l., *B. miyamotoi*, *A. phagocytophilum*, *Rickettsia helvetica*, *B. divergens*, *B. microti*, *Babesia venatorum* and TBEV (Rizzoli et al. 2014). With an estimate of 60,000–100,000 total and 7500 hospitalised cases annually, Lyme borreliosis is regarded as the most frequent human TBD in Germany (Lohr et al. 2015). However, since only certain manifestations of Lyme borreliosis are reportable in some, but not all, federal states, this number may be inaccurate (Lohr et al. 2015). In contrast, TBE is notifiable in all parts of Germany and annual case numbers ranged between 195 and 584 in the period 2001–2019 (Robert Koch-Institut 2020). Less is known regarding other TBDs in Germany, but human cases of neoehrlichiosis (von Loewenich et al. 2010) and babesiosis due to *B. venatorum* (Häselbarth et al. 2007) and *B. microti* (Hildebrandt et al. 2007) have been reported during the past decades. With regard to *Rickettsia* spp., *R. helvetica* is the predominant species, but *Rickettsia monacensis*, *Rickettsia slovaca* and *Rickettsia raoultii* also occur in Germany (Dobler and Pfeffer 2012). In addition, travellers returning from other countries may be infected with non-endemic tick-borne pathogens, e.g. *Rickettsia africae* (Antal et al. 2013), necessitating appropriate diagnostic possibilities.

Regarding veterinary medicine, no estimates of annual TBD incidence exist. However, granulocytic anaplasmosis is regarded as the most important TBD in dogs, whereas Lyme borreliosis may be overdiagnosed (Gerber et al. 2009). Furthermore, *A. phagocytophilum* is relevant as the causative agent of granulocytic anaplasmosis in horses (Silaghi et al. 2011) and tick-borne fever in ruminants (Nieder et al. 2012). In ruminants, redwater fever due to *B. divergens* occurs sporadically and may lead to significant mortality in naïve cattle herds (Springer et al. 2020). In addition, sporadic clinical cases of TBE have been described in German dogs (Reiner and Fischer 1998; Saenger et al. 2013).

Commercially available diagnostic kits, taking Germany as an example, were identified by Google Search using combinations of the following keywords: *Anaplasma*, *Babesia*, *Borrelia*, *Rickettsia*, *Ehrlichia*, TBE, FSME, IgG, IgM, PCR, ELISA, ELISPOT, IFAT, serology and kit. Furthermore, a list of available diagnostic tests for *B. burgdorferi* s.l. and TBEV was obtained from the German National Reference Center for Borrelia and the German National Consiliary Laboratory for TBEV, respectively. In addition, the German Diagnostics Industry Association contributed a list of relevant manufacturers, whose websites were searched for relevant test kits.

In Table 4, the relative quantities of commercially available diagnostic test kits for human vs. veterinary use for each pathogen are shown. Only tests designed for patient samples were included, i.e. tests for pathogen detection in ticks were not considered, since a positive result in the detached tick is not a reliable indicator of human or animal infection. In-house tests and research-use only tests were also not considered. No absolute numbers are shown, because we cannot guarantee that the search was exhaustive and, furthermore, the market is subject to frequent changes.

**Table 4:** Relative quantity of commercially available diagnostic tests for zoonotic tick-borne pathogens in Germany

Pathogen	Nucleic acid detection		Antibody detection		Other tests (e.g. ELISPOT)	
	For veterinary (vet.) use	For human use	For vet. use	For human use	For vet. use	For human use
*Babesia divergens*	−	+	+ (IgG: +, IgM: −, IgG/IgM: −)	−	−	−
*Babesia microti*	−	+	+ (IgG: +, IgM: −, IgG/IgM: −)	+ (IgG: +, IgM: −, IgG/IgM: −)	−	+
*Babesia venatorum*	−	+	−	−	−	−
*Bartonella henselae*^1^	−	+	+ (IgG: +, IgM: −, IgG/IgM: −)	+ (IgG: +, IgM: +, IgG/IgM: −)	−	+
*Borrelia burgdorferi* s.l.	+	++	++ (IgG: ++, IgM: +, IgG/IgM: +)	+++ (IgG: +++, IgM: +++, IgG/IgM: ++)	+	+
*Borrelia miyamotoi*	−	−	−	−	−	+
*Coxiella burnetii*	++	−	++ (IgG: ++, IgM: +, IgG/IgM: −)	+++ (IgG: ++, IgM: ++, IgG/IgM: −)	−	−
*Francisella tularensis*	−	+	+ (IgG: +, IgM: −, IgG/IgM: −)	++ (IgG: ++, IgM: +, IgG/IgM: −)	−	−
*Anaplasma phagocytophilum*	+	+	++ (IgG: ++, IgM: −, IgG/IgM: −)	++ (IgG: ++, IgM: +, IgG/IgM: −)	−	
*Ehrlichia* spp.	+	−	++ (IgG: ++, IgM: −, IgG/IgM: −)	+ (IgG: +, IgM: +, IgG/IgM: −)	−	+
*Neoehrlichia mikurensis*	−	−	−	−	−	−
*Rickettsia* spp.	−	++	++ (IgG: ++, IgM: −, IgG/IgM: −)	++ (IgG: ++, IgM: ++, IgG/IgM: −)	−	−
Tick-borne encephalitis virus	+	+	+ (IgG: +, IgM: −, IgG/IgM: −)	+++ (IgG: ++, IgM: ++, IgG/IgM: +)	−	−

+++, > 20 kits on the market; ++, 6–20 kits on the market; +, ≤ 5 kits on the market; −, no marketed kits found

^1^Vector competence of ticks for *B. henselae* not proven

Results indicate that a multitude of serologic kits and, to a lesser extent, nucleic acid detection kits are available for diagnosis of Lyme borreliosis and TBE in humans in Germany (Table 4). A rather large number of kits was also retrieved for Lyme borreliosis in animals, but only few for TBE, although domestic animals have proven useful as sentinels of human disease risk (Imhoff et al. 2015). In addition, most veterinary serology kits for *B. burgdorferi* s.l. detect IgG antibodies only, whereas an equal amount of IgG and IgM tests exists for humans. This can be explained by the fact that animals usually do not develop acute disease after *B. burgdorferi* s.l. exposure, and IgM testing is thus not recommended (Littman et al. 2018). However, a positive IgG titre is not an indicator of active infection and it can be extremely difficult to determine whether clinical disease in animals is actually due to *Borrelia* infection (Divers 2013; Littman et al. 2018). To reduce unnecessary antibiotic use, reliable tests indicative of active infection would be extremely helpful in both disciplines. As highlighted above, IgG avidity testing or improved PCR procedures, such as digital PCR, could be promising approaches.

Regarding *A. phagocytophilum*, a similar amount of serologic as well as nucleic acid detection kits was identified for the human medical as well as the veterinary market, probably because *A. phagocytophilum* plays an important role in veterinary medicine, affecting several species as described above. The number of available veterinary serology kits for *Ehrlichia* spp. even exceeded the amount available for use in human medicine, but no direct detection kits for *Ehrlichia* spp. were identified for veterinary use. Ticks transmitting zoonotic *Ehrlichia* spp. (*Rhipicephalus sanguineus* s.l., *A. americanum*) are not endemic in Germany; thus, ehrlichioses are only relevant as imported diseases. *E. canis* is a major threat to canine health worldwide (Rar and Golovljova 2011), including in Mediterranean Europe from where many dogs are imported to Germany and other Central or Northern European countries. In contrast, human ehrlichiosis cases are rather rare, occurring mainly in North America (Rar and Golovljova 2011), and are thus more rarely imported to Germany than canine cases. Consequently, the available veterinary kits were mostly designed for *E. canis* antibody detection.

In contrast, only few kits for the diagnosis of rickettsioses in animals were identified, probably because it is unknown whether *Rickettsia* spp. cause disease in animals, with the exception of *R. conorii* and *R. rickettsii* in dogs (Keenan et al. 1977; Solano-Gallego et al. 2006). Neither of these species is endemic in Germany (Dobler and Pfeffer 2012). Regarding humans, several serologic as well as direct detection kits for tick-borne *Rickettsia* spp. were identified, mainly designed for *R. rickettsii* and *R. conorii* detection.

Particularly few diagnostic kits were identified regarding infections with zoonotic *Babesia* spp., both in the human medical and in the veterinary sector. This may be due to the fact that *Babesia* infections are often diagnosed by blood smears and/or in-house PCR tests in acute cases. However, blood smears have a limited sensitivity when parasitaemia is low or limited specificity when parasite morphology has been altered due to refrigeration prior to blood smear preparation (Cursino-Santos et al. 2014). In addition, many human babesiosis cases in immunocompetent individuals might be overlooked when symptoms are mild, which represents a problem regarding blood transfusions, for example (Hildebrandt et al. 2008; Ord and Lobo 2015). In the veterinary field, a recent outbreak of bovine babesiosis (*B. divergens*) in Germany has shown that mortality rates and the subsequent economic impact may be high if diagnosis is delayed (Springer et al. 2020). Therefore, sensitive, easy-to-use and rapid diagnostic tools for zoonotic *Babesia* spp. are needed. Recently, an immunochromatographic test based on a recombinant *B. microti* surface antigen showed promising results in experimentally infected mice (Cai et al. 2018).

Regarding *B. miyamotoi* and *N. mikurensis*, which have only recently been identified as human and, possibly, veterinary pathogens (Diniz et al. 2011; Platonov et al. 2011; Welinder-Olsson et al. 2010), no commercially available kits were identified at all, except for one ELISPOT kit designed for *B. miyamotoi*. In general, only few ELISPOT assays are currently available in Germany, reflecting the fact that their utility is controversially discussed. Identified tests included EPISPOTS for detecting cellular immunity against *B. burgdorferi* s.l. in humans, horses and dogs, as well as against *B. miyamotoi*, *B. microti*, *Ehrlichia* spp. and *Bartonella henselae* in humans.

For *Bartonella henselae*, *C. burnetii* and *F. tularensis*, tick-borne transmission plays a minor role. Several diagnostic kits were identified for *C. burnetii* for both disciplines, as this pathogen is economically important as a cause of abortions in ruminants as well as from a public health perspective (Duron et al. 2015). In contrast, identified diagnostic kits for *F. tularensis* were mainly for human use, as symptomatic infections in domestic animals are limited to cats and rabbits (Telford III and Goethert 2020).

## Conclusions

Human and animal health are closely linked by ticks acting as vectors for zoonotic pathogens, making tick-borne diseases excellent examples of the One Health concept. Animals are either clinically affected by the same tick-borne pathogens as humans and/or play a role in tick cycle maintenance and as reservoirs or sentinel pathogen hosts. Using the German market as an example, several gaps in commercial availability of diagnostic tests for zoonotic tick-borne pathogens were identified. Regarding *B. burgdorferi* s.l., sensitive tests indicative of active infection would be useful to limit unnecessary or overuse of antibiotics in human as well as veterinary medicine. Furthermore, there is a need for rapid and sensitive diagnostic tools for zoonotic *Babesia* spp. infections in both disciplines. Recently emerged tick-borne pathogens, such as *N. mikurensis* and *B. miyamotoi*, open up further opportunities for collaboration, since no standardised tests for these pathogens are yet commercially available. Test development for these pathogens could save substantial time and effort for the benefit of both human and animal health.
